# A Navigation System for the Visually Impaired: A Fusion of Vision and Depth Sensor

**DOI:** 10.1155/2015/479857

**Published:** 2015-08-18

**Authors:** Nadia Kanwal, Erkan Bostanci, Keith Currie, Adrian F. Clark

**Affiliations:** ^1^Department of Computer Science, Lahore College for Women University, Jail Road, Lahore 54000, Pakistan; ^2^Computer Engineering Department, Ankara University, Gölbaşı, 06830 Ankara, Turkey; ^3^Department of Literature, Film and Theater Studies, University of Essex, Colchester, Essex CO4 3SQ, UK; ^4^School of Computer Science and Electronic Engineering, University of Essex, Colchester, Essex CO4 3SQ, UK

## Abstract

For a number of years, scientists have been trying to develop aids that can make visually impaired people more independent and aware of their surroundings. Computer-based automatic navigation tools are one example of this, motivated by the increasing miniaturization of electronics and the improvement in processing power and sensing capabilities. This paper presents a complete navigation system based on low cost and physically unobtrusive sensors such as a camera and an infrared sensor. The system is based around corners and depth values from Kinect's infrared sensor. Obstacles are found in images from a camera using corner detection, while input from the depth sensor provides the corresponding distance. The combination is both efficient and robust. The system not only identifies hurdles but also suggests a safe path (if available) to the left or right side and tells the user to stop, move left, or move right. The system has been tested in real time by both blindfolded and blind people at different indoor and outdoor locations, demonstrating that it operates adequately.

## 1. Introduction

A navigation system for a person with visual impairment involves identifying the layout of the 3D space around them and then helping them negotiate their way around obstacles* en route* to their destination. The traditional aid for this is a white cane, swept from side to side in front of the person; however, computer technology has the potential to provide less obtrusive and longer-range aids. To acquire this information, a variety of sensors could be used; some of the commonly used ones are summarized in Table 1. Sonar and laser strippers are able to provide the distance to objects and therefore have been previously used in developing automated navigation solutions for robots and humans. However, all of these sensors have limitations, such as the poor angular resolution of sonar because of its wide beamwidth [1] and the cost of laser strippers. There is no clear winner in the choice of sensor; but the cheapness, small size, and ease of integration of cameras make them attractive if one can overcome the difficulties of segmenting objects and so forth. Arguably, the most appealing way to produce such a system is to use a body-mounted video camera combined with computer vision. As a single camera is unable to detect distance, a pair of cameras is normally used as they allow computational stereo to determine the distance to obstacles/humans [2].

An even more attractive solution would be to detect distance directly, a capability offered by the Microsoft Kinect and similar devices. Though developed initially as a gaming input device for the Xbox 360, the Kinect has become popular among vision researchers because of both its low cost and the availability of software to acquire and work with data from it [3]. The Kinect features both a conventional colour camera and a depth sensor, the latter operating by projecting an infrared structured light pattern and using a camera sensitive in the infrared to capture where it falls on objects and then comparing the captured pattern with a reference one to determine disparity and hence depth. In principle, one should be able to determine the placements of obstacles using only the depth sensor; however, in practice, there are distance estimation and structural and noise problems [4] which make it difficult to obtain distance information from some objects because they are not reflective at infrared wavelengths. For example, occlusion results in shadows, creating blind spots which cannot be used to obtain distances. Nevertheless, researchers have already employed the Kinect to produce navigation systems [5–8]. In [5], the Kinect was integrated with an array of vibrotactile elements to build an assistant for blind navigation. Similarly, [7, 8] used the Kinect to identify the distances to objects, though in that work the sensor is static and the system uses only the distance sensor's data to determine an obstacle's distance from user.

This research also uses a Kinect sensor but processes data acquired by both its depth sensor and its camera. An attempt has been made to utilize the strength of both sensors by detecting obstacles using corner detection and then estimating their distance using depth map of the same scene. The focus is to overcome the limitations of vision algorithms in detecting and matching features subject to geometric and photometric transformations [9].

The rest of the paper is structured as follows.  Section 2 introduces Kinect and its working and its sensors' calibration and provides context concerning the computer vision processing and reviews briefly some local image features that can be used for vision-based navigation. Furthermore, it presents the complete, working navigation system, results from which are presented in Section 3. The accuracy of this kind of systems is more important than its speed; hence, the system is tested on two people, one blindfolded and the other visually impaired.  Section 3.3 describes how a blindfolded person responded to the system's guidance and then how well the system guided a person with visual impairment. The section also includes reaction time analysis of blindfolded and blind person. Based on this feedback, Section 4 examines the current limitations of the system and how these can be addressed in the future. Finally, Section 5 gives some concluding remarks.

## 2. Material and Methods

### 2.1. Microsoft Kinect for Xbox 360

**Figure 1:**
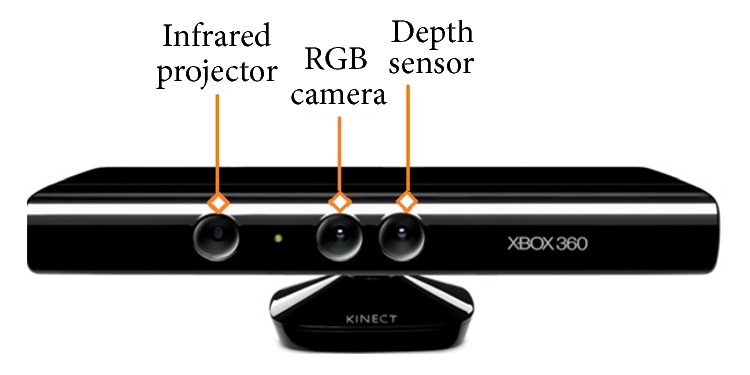
Microsoft Kinect for Xbox 360.

The Kinect was a sensor developed by Microsoft to capture human motion for the Xbox 360 gaming console, shown in Figure 1. It has two sensors, a camera, and an infrared sensor designed to estimate a user's motion while playing games. Soon after its SDK release, researchers have used it not only for developing 3D games but also for a number of other interesting applications, including face tracking, picture frame, and digital mirror [3].

However, one has to be careful while using Kinect to develop navigation systems as the sensor is not flawless and has some limitations. For example, it projects infrared light and uses its reflection to calculate the depth image. These reflections may sometimes be sensed incorrectly or may be absent due to irregular or nonreflective surfaces. Similarly, the infrared light can be swamped by strong light sources such as sunlight; and, most importantly, depth values from the sensors are not stable even if it is stationary [4]. These inaccurate depth data reduce the accuracy of the system. Furthermore, reflections from the complete field of view, for example, floor, are also obtained, so obstacle detection becomes difficult without understanding the image content.

*Calibrating the Kinect Sensor*. The Kinect sensors are located at a short distance apart, so the first step in using the Kinect in a vision system is to calibrate them individually and to determine their separation; the latter is particularly important for this application as poor measurement of the separation is manifested as disparity between the colour and depth sensors.

To calibrate the Kinect, one can either use a dedicated Matlab toolbox [10] or identify corners in both the colour and depth images as described in [11, 12]. The latter method was used here as it is similar to the algorithms used in the navigation system, allowing it to be integrated into its initialisation phase rather than as a once-only, offline calibration. To perform the calibration, one captures images of a calibration target using both Kinect sensors and then identifies the same corner points in the colour and distance images manually. From these values, camera distortion coefficients and transformation matrices can be calculated using the process described in [11, 12]. The various parameters involved are summarised in the following formulae, while their calibrated parameters are shown in Table 2:

(1) SensorRGB=fx0tx0fyty001,SensorDepth=fxd0txd0fydtyd001,Distortion Coefficients=k1k2p1p2k3.

The rotation matrix _*R* and translation matrix _*T* that result from these are

(2) _R=9.99×10−11.26×10−3−1.75×10−2−1.48×10−39.99×10−3−1.23×10−21.75×10−21.23×10−29.99×10−1,_T=1.9985×10−2−7.4424×10−4−1.0917×10−2.

The Kinect distance sensor returns a depth “image” in which each pixel corresponds to the distance of the object in a colour image pixel, with some translation and rotation due to the physical separation of the two sensors on the device. Therefore, to find the depth of colour image pixels, one projects each depth image point in real world by calculating each colour image pixel's 3D coordinates using

(3) $$\begin{array}{c} \text{\_}P_{\text{3D}}=\left(\begin{array}{c} p_{x}\\ p_{y}\\ p_{z} \end{array}\right)=\left(\begin{array}{c} xt_{xd}\frac{d_{m}}{f_{xd}}\\ yt_{yd}\frac{d_{m}}{f_{yd}}\\ d_{m} \end{array}\right), \end{array}$$

where *d*_*m*_ is the distance in metres calculated using

(4) $$\begin{array}{c} d_{m}=0.1236*\tan\left(\;\frac{D}{2842.5}\;\right)+1.1863, \end{array}$$

where *D* is the raw depth value obtained from the Kinect. This conversion is important because the rotation and translation matrices _*R* and _*T* are calculated in metres in the real world. From these 3D coordinates, 2D projections onto the colour image can be computed using

(5) P2Dpx2Dpy2Dpz=_R_P3D+_T,Px2DPx2DPz,Py2DPy2DPz,

so that the colour image pixel corresponding to a depth estimation can be determined. To speed up the process, a 2D lookup table can be generated, indexed on 2D projections of depth image pixels.

### 2.2. Low-Level Image Features for Navigation Systems

Image understanding and matching have been long-standing, important research areas in devising safe navigation systems for both robots and humans [13]. Vision-based navigation systems commonly use feature-based matching in video frames. These image features can be blobs, edges, corners, or regions. A blob is an image area with a significant intensity difference from its neighbourhood, for example, a dark spot in a bright region or a bright spot in a dark region [14]. An edge is the boundary between image regions, usually identified in terms of colour or intensity difference, and is important when segmenting image regions [15]. Similarly, image regions are also used as features to be matched for segmentation tasks [16]. Finally, corner points are the image areas that correspond to sharp changes in the direction of boundaries [17].

**Figure 2:**
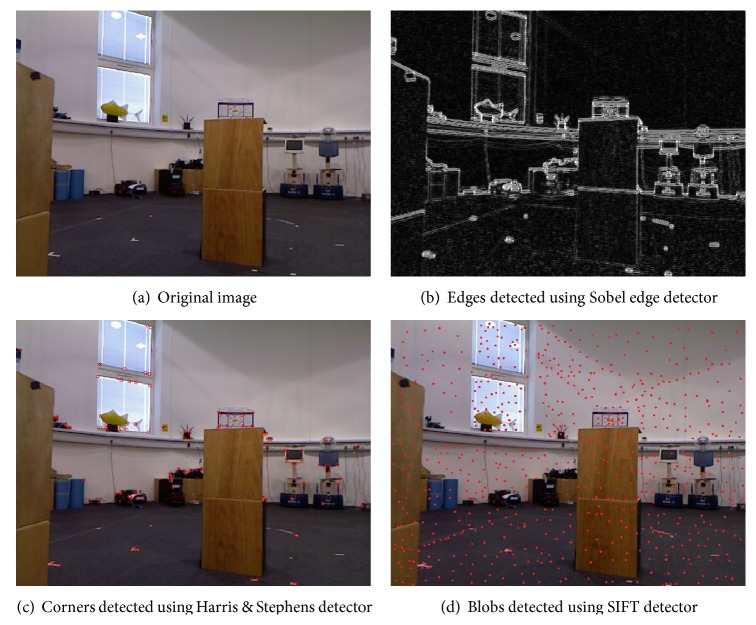
Results of local image feature detectors that can be used to develop a vision-based navigation system.

Although any of these local image features can be used for this application, the sensitivity of infrared sensor to reflective materials means that the use of blobs for depth calculation can degrade the system's performance, particularly outdoors. Furthermore, extracting regions takes more time than corners but gives less information about image content.  Figure 2 shows that if the detected features are edges or blobs, the result is every textured area in the image. This might be useful for applications where identification of the whole image content is required, such as segmentation, panorama stitching, and homography estimation; however, in this navigation application, meaningful image areas such as obstacles are important, and the result in Figure 2(c) shows that the corner points identify important image content.

Furthermore, corners are particularly attractive because they are fast to compute and lie on the boundaries of obstacles. Consequently, corner points have been chosen to find image locations that can correspond to obstacles.

After detecting corner points in images, matching them in subsequent video frames is required for a navigation or tracking application. The best-known methods in the literature for tracking features are homography-based matching [18], visual odometry, optical flow, and particle filtering. Visual SLAM [19] and related systems have also been developed using matching of local image features [20] or corners [21]. Despite all these efforts, there are limitations of vision algorithms to detect and match features under geometric and photometric transformations [9], resulting in low repeatability scores and unstable responses at different scales, which makes it difficult to obtain consistent, continuous information for safe navigation. This research explores whether one possible solution is to combine the image information from different sensors, such as the Kinect camera and its infrared sensor.

### 2.3. A Kinect-Based Navigation System

**Figure 3:**
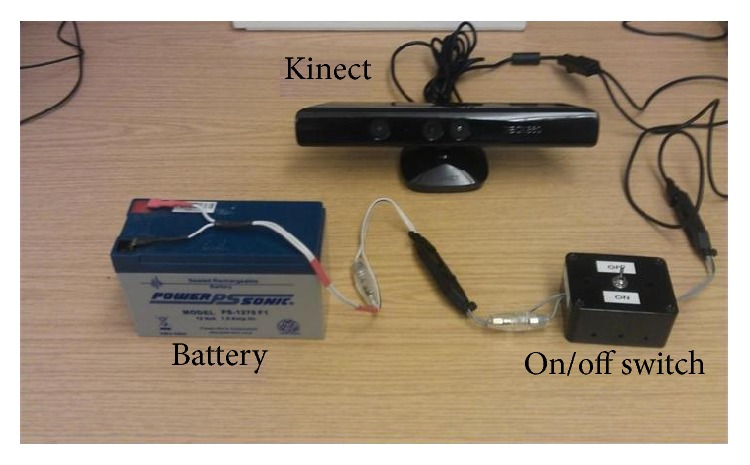
Components of the navigation system, excluding the laptop or equivalent on which all processing is performed.

The navigation system described here requires a standard Kinect sensor, a battery, and a laptop/processor, all of which are carried in a backpack or shoulder bag by the user. The Kinect is powered by a sealed lead acid 12 V, 7 A battery shown in Figure 3, the output of which is fed through a DC to DC converter to ensure a stable 12 V supply. The capacity of the battery is enough to power both Kinect and a potable CPU for 3 hours. The Kinect sensor is carried in front of the user using a strap around their neck; though manageable, this is bulky and the authors would expect a production system to repackage the sensors into a unit that is physically smaller and easier to manage, perhaps shoulder mounted.

**Figure 4:**
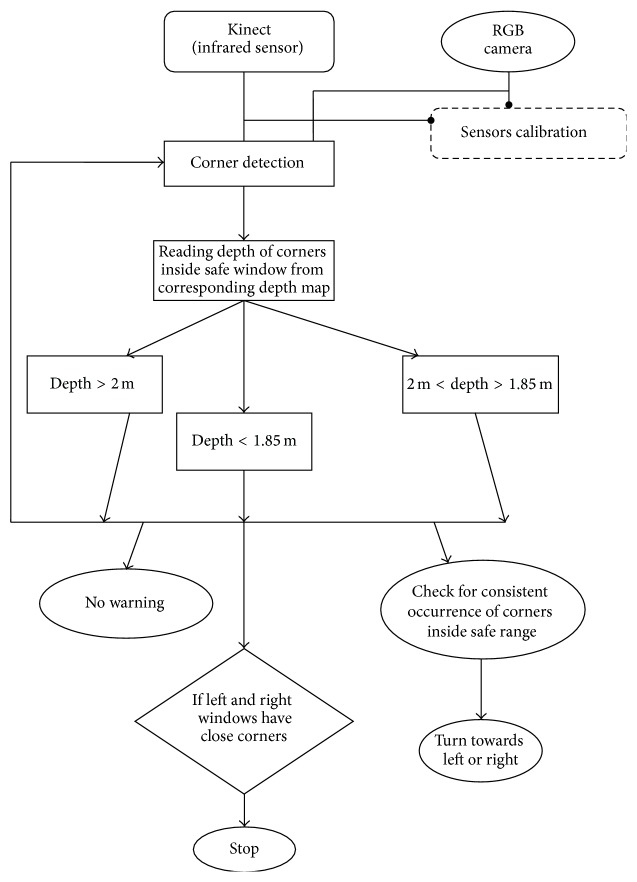
Algorithm describing the conversion of input data into sensible verbal feed for visually impaired person.

*Software Issues*.  Figure 4 describes the algorithms developed. The proposed system uses the Harris & Stephens corner detector to find corners in RGB images acquired by Kinect's camera, with their depth being obtained from the depth lookup table constructed from 2D projections of depth image pixels using the measurements described in Section 2.1.

**Figure 5:**
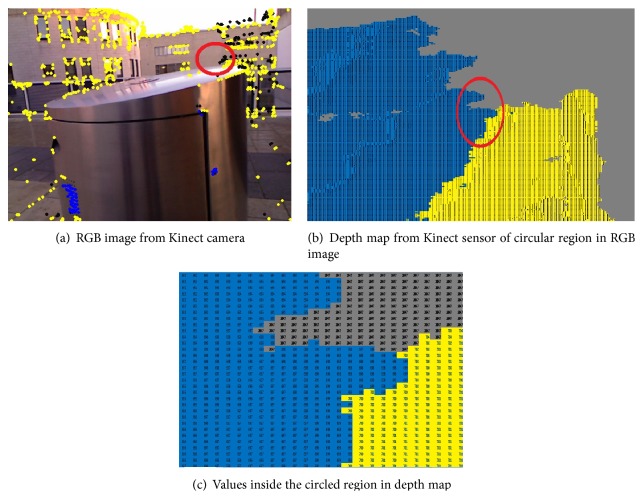
Kinect output: an RGB image and its corresponding depth map; colours in the depth estimated map represent distance from the sensor based on distance threshold: green color shows possible hazard, whereas blue color represents potential hazard and black points are the areas from which the sensor is unable to get sensible depth and the values are 2047.

It is important to bear in mind that not all values from the Kinect distance sensor are correct due to the problems alluded to in Section 1.  Figure 5 shows depth map of an RGB image where black pixels are no depth areas and, therefore, if the detected corner point is one of these pixels then its depth value cannot be used for distance calculation. To overcome this problem, the system searches for a sensible depth value in a 10 × 10-pixel neighbourhood around colour image corners.

**Figure 6:**
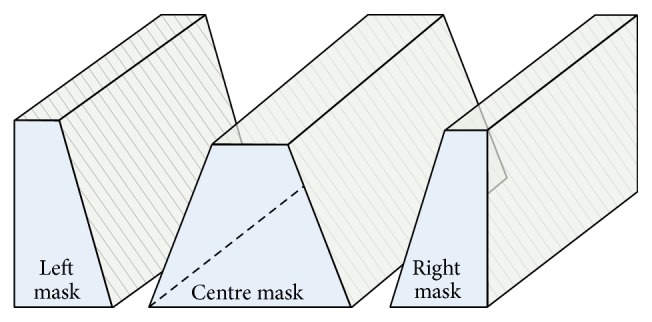
Image masks showing which parts of the colour and depth images are processed (in white) for a user travelling straight ahead (center mask). These white regions are termed “safe navigation regions.” The equivalent masks used to assess whether to turn to the left or right are shown to the sides of the center mask.

To enable a partially sighted person to navigate freely, the entire image is divided into regions (masks), shown in Figure 6. The white pixels in each region need to be obstacle-free for the person to move in that direction, and there are separate mask images for (from left to right in the figure) turning to the left, walking straight ahead, and turning to the right. These white regions are termed “safe navigation regions.” The width of the safe navigation region in the centre mask image is set to match the area which an average sized person occupies in real world coordinates, so it is wider near the person and narrower at greater distances. The proportions of three masks in the image are 80%, 10%, and 10% for central, left, and right masks, respectively. The minimum depth of each window is kept the same, that is, 1.85 m more than the average length of a white stick (1.42 m), and within the maximum working range of infrared sensor.

*Processing*. The system checks for two types of obstacles, those that can completely block progress and smaller ones such as chairs that can be avoided by changing direction. For the former, the image feature detector may fail if the surface is featureless so the system checks all depth values inside the safe navigation region (Figure 6) and if the mean depth becomes less than a threshold the user is notified to stop using voice synthesis. Otherwise, the feature detector finds corner points and, depending on the depth of corners, warnings may be issued to change directions to the right or left. The system employs the processed depth (*I*_*d*_) and colour (*I*_*c*_) images from the Kinect in a three-stage processing algorithm described in the following paragraphs:

(1) *Preprocessing*. Calibration parameters are applied to both input images (*I*_*d*_ and *I*_*c*_) so that distortions can be removed from them. Then, corner points are found in the colour image while the depth image's data are converted into meters, from which the 2D projection of all points is found and the 2D lookup table described in Section 2.1 calculated. For fast processing, this stage works in separate threads because calculations on *I*_*c*_ and *I*_*d*_ are independent of each other.
(2) *Navigation Processing*. The central safe navigation region is scanned for wall-like obstacles by averaging the depth values. If the mean depth is less than a threshold (1.85 m in this work, based on suggestions by white cane user), the system activates the alarm-generation module and warns the user to stop. Otherwise, the system looks for each corner point's depth within the central safe navigation region. If one or more corner points appear to be close to the person (their distance is less than the threshold), the system starts counting frames for which these points remain a hazard. The walking speed of a blind person is typically 2 to 3 miles/hour (0.6 m/s) [22] so if hazardous corner pixels remain in the active region for five frames, it checks the right and left safe navigation regions for a clear route, selecting the one with no wall-like obstacle and where the nearest corners are further from the user. Once the left or right window is selected, the system initiates the alarm-generation module to inform the user. If no obstacle is found, the system remains quiet.
(3) *Alarm*. If an alarm is triggered during processing, voice synthesis is used to alert the user, generating, for example, “*stop*,” if there is an obstacle in the way. For a left or right movement, the system generates “*left*” or “*right*.”

**Figure 7:**
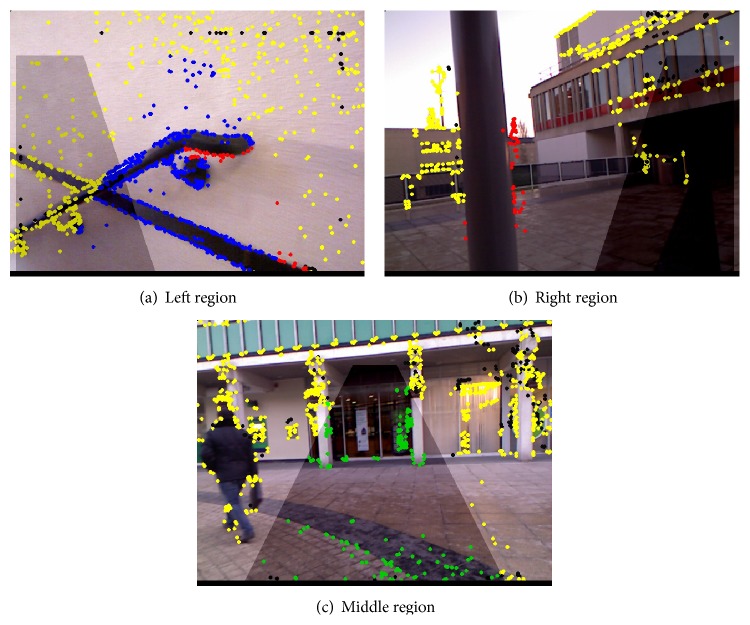
Navigation from analysing data in the image masks of Figure 6. In (a), the user is asked to move to the left; in (b), the user is given an alarm and encouraged to move to the right; and in (c), the user can move forward.

Figure 7 illustrates three different scenarios. Corner points are presented in five different colours:

(i) *Yellow* points are nonhazard corner outside the safe navigation regions.
(ii) *Green* points indicate potential hazards (*d*_*m*_ > 2 m inside the safe navigation regions).
(iii) *Blue* points are potential hazards (2.0 m > *d*_*m*_ > 1.85 m inside the safe navigation regions).
(iv) *Red* points are hazards (*d*_*m*_ < 1.85 m inside the safe navigation regions).
(v) *Black* pixels indicate a blind spot in depth image.

In Figure 7(c), the system does not find any wall or obstacle so all the corner points are green and there will be no warning to the user. In Figure 7(a), there are some points which lie inside central safe navigation region and cross the safe distance threshold (red-coloured corners), so the system checks right and left safe navigation regions and finds the left one to be the safer and so prompts the user to move left. Conversely, Figure 7(b) shows where, after finding an obstacle in the central safe navigation region, the system identifies the right as being safer and prompts the user to move that way.

Although Kinect sensor is attached to the subject's body and therefore does not remain stationary during motion, however, due to the installation of both sensors at the same axis, their calibration compensates the jittering effects.

## 3. System Testing

The proposed system aims to provide navigation assistance to visually impaired people, so testing by its probable users was crucial. To circumvent any potential problems, two test cases have been used. In the first case, a blindfolded person was asked to use the system to navigate in an unknown environment containing obstacles, while in the second case a visually impaired person was requested to try the system and assess its accuracy in real time.

**Figure 8:**
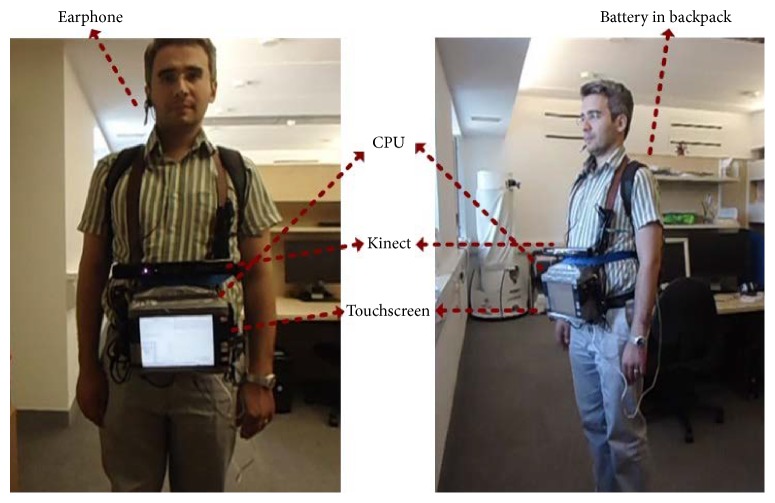
Hardware setup for testing the system.

Figure 8 shows the hardware setup used to perform these experiments. The durations of the experiments were kept short because the equipment was bulky and heavy, though a belt through waist was also used to provide some extra support and to distribute the equipment's weight evenly. The Kinect and a touchscreen monitor were mounted on a portable CPU and all of the three devices (CPU, Kinect, and monitor) were powered by the battery shown in Figure 3, which was placed in a backpack. The results of the experiments are given below.

### 3.1. Test Case 1: A Blindfolded User

**Figure 9:**
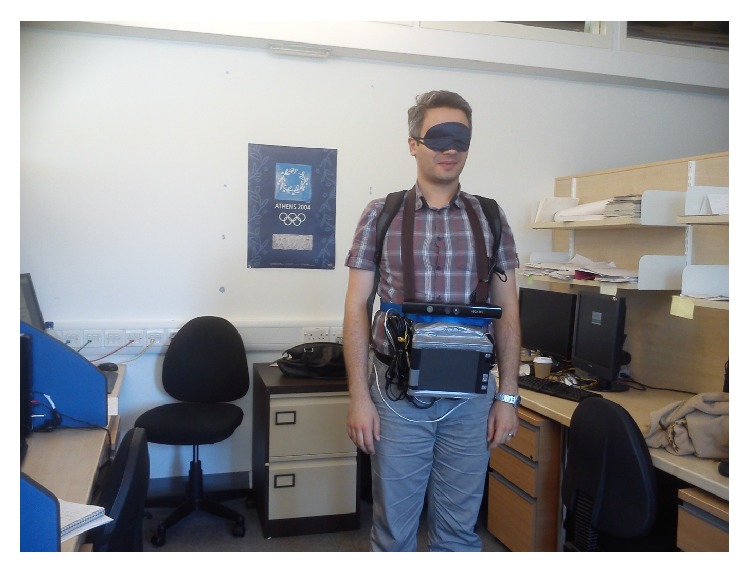
System testing by a blindfolded person.

Figure 9 shows how a person with proper visual sense tries to sense the environment using the Kinect-based system instead of his own eyes. The system was required to be calibrated according to the user's height, setting the distance threshold and the number of frames per response to match their walking speed.

**Figure 10:**
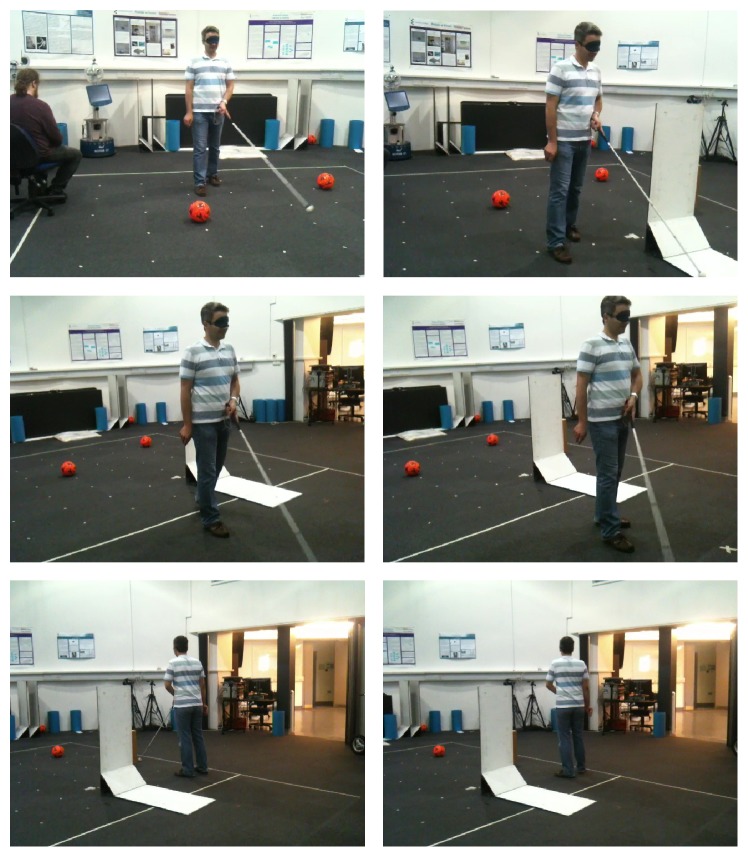
A blindfolded person using white cane as mobility aid.

The user was able to move in different indoor and outdoor locations with good confidence of the system's guidance. However, as expected, the user found the system response time to be slow. This appears to be because a person with good visual sense does not naturally walk as slowly as a blind person walks, and has difficulty walking at that pace. This became quite obvious when the same user was asked to use a white cane as mobility assistant (shown in Figure 10). His stress level was high during this experiment, with the user keeping his other arm close to his body to save himself from collisions. Similarly, the walking speed was significantly slower, showing that using an unfamiliar mobility aid affects the user's confidence.

### 3.2. Test Case 2: A Visually Impaired User

**Figure 11:**
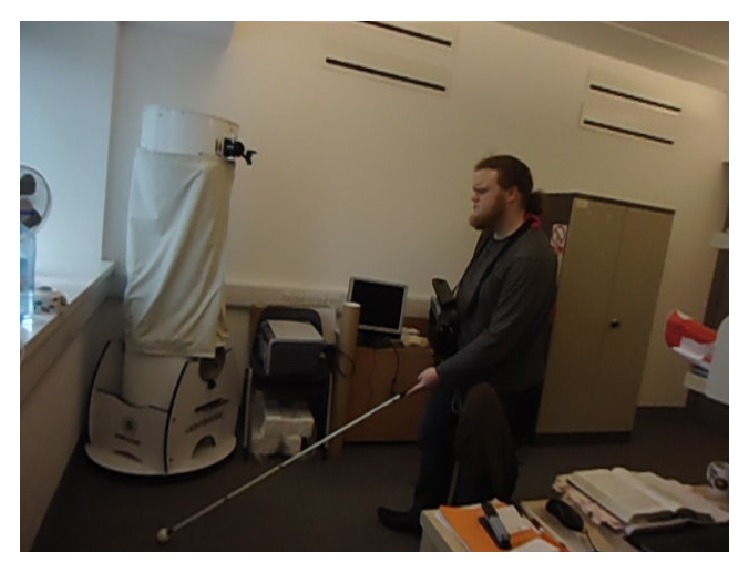
Calibrating Kinect-aided navigation system by letting a visually impaired person walk using it along with white cane.

*Dr. Keith Currie*, a born blind man, participated in the experiment to test the Kinect-aided navigation system. Keith was enthusiastic and optimistic about the whole experiment because of his interest in such kind of mobility aids. The authors admire his courage and bravery for testing a development system.  Figure 11 shows him using the system along with a white cane, allowing him to familiarize himself with the system and to calibrate it according to his needs.

**Figure 12:**
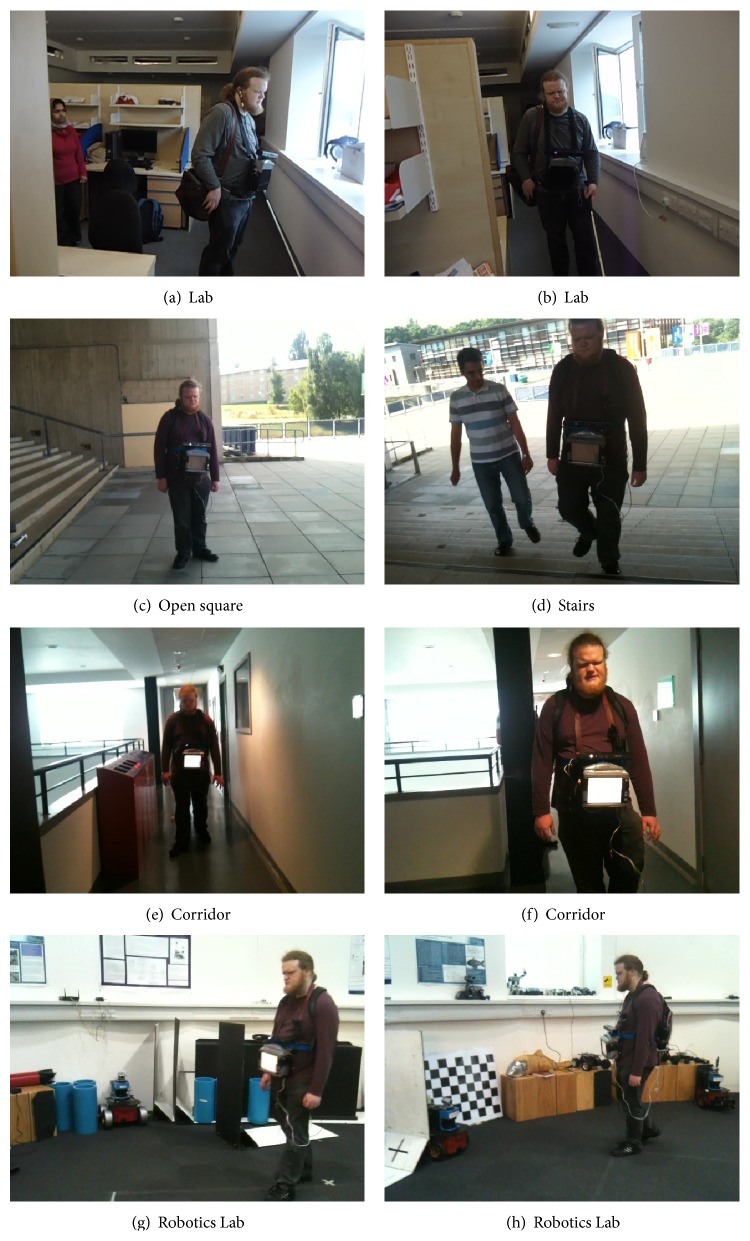
*Keith Curie* walking at different locations using Kinect-aided navigation system.

In order to increase his comfort level with the system, the system was tuned to communicate at every alternate frame, by saying “*ok*” during first attempt, even if no obstacle was detected. In this way, the user was able to know that the system was working and he can take further steps without bumping into an obstacle. Furthermore, letting him use white cane along with the automated system helped identify the best distance threshold for a blind person. Initially, the distance threshold was set to 0.3 m, less than his white cane which was approximately 1.42 m long. As a consequence, Keith was able to sense obstacles before the system and therefore did not find it helpful. However, after changing the distance threshold to 1.85 m, the system was able to notify the user before the white cane, improving its usefulness.  Figure 12 presents the locations where Keith tested the system: different indoor and outdoor locations.

### 3.3. Analysis of the Proposed System

**Figure 13:**
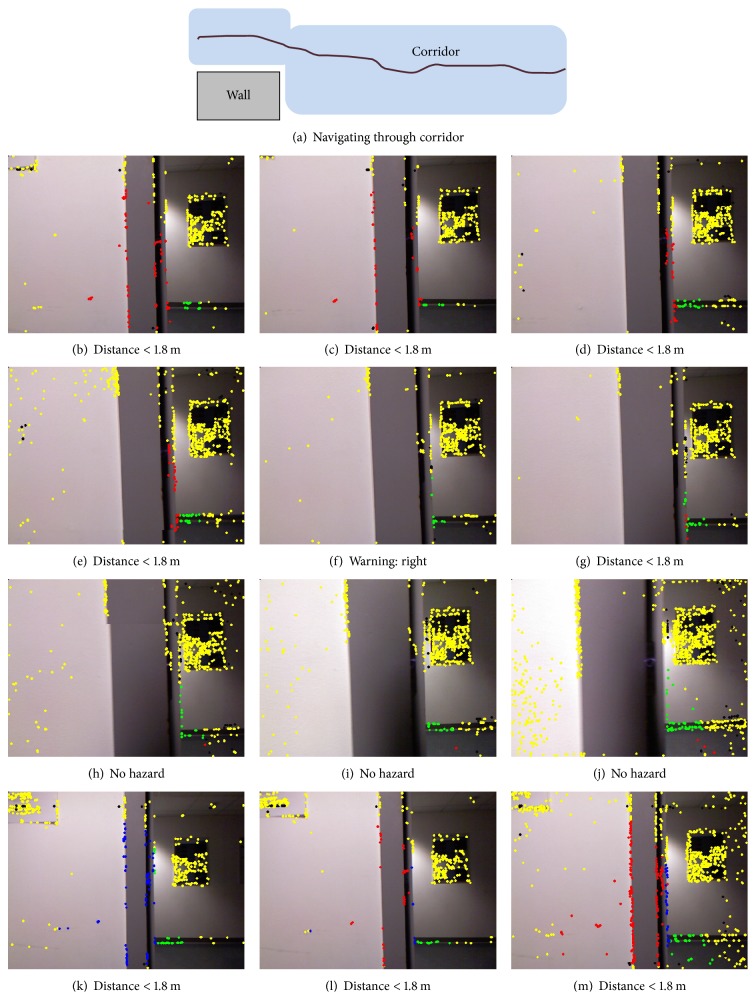
The navigation system's visual output on an indoor video sequence of walking through a corridor coming in front of a wall. Here, (a) describes the navigational path by the subject (blind person). The system counts frames for consistent presence of hazard (b) till (e) and then generates voice signal “right” on frame (f). Similarly, the system starts counts on frame (g) but then no warning is generated because it loses red points in subsequent frames till frame (l).

**Figure 14 fig14:**
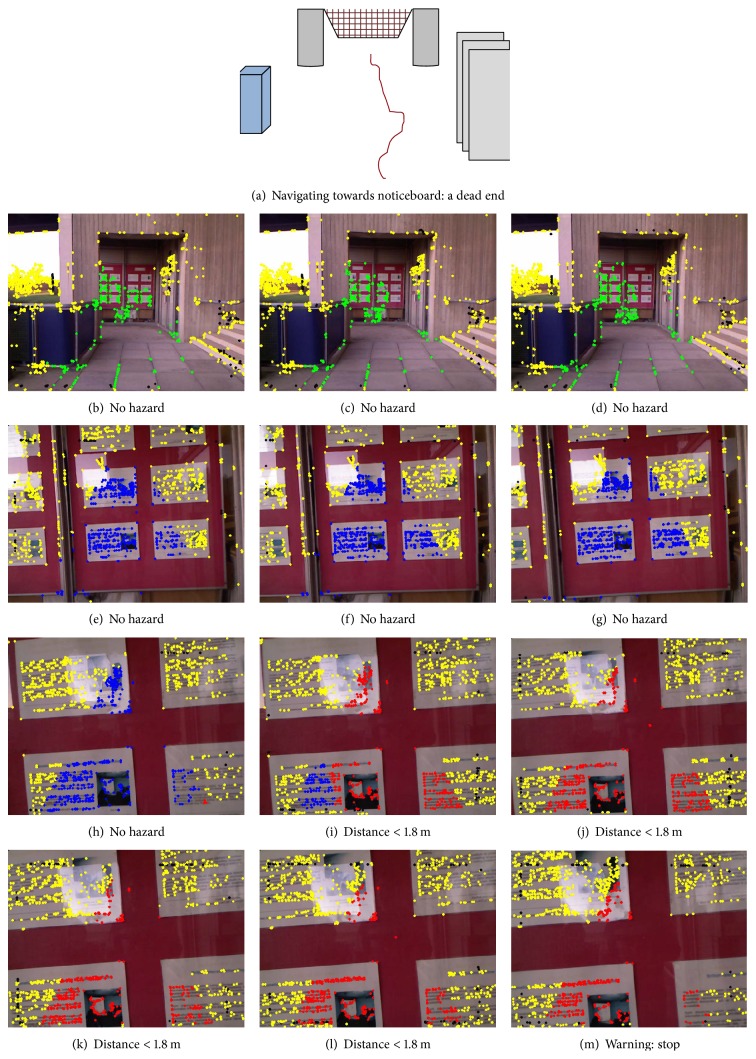
The navigation system's visual output on an outdoor video sequence, walking towards a wall with noticeboard. (a) presents the complete navigation of the subject (blind person). Images in (b–m) show system's processing. The system finds red points in frame (i) and so starts counting and generates verbal warning to stop on frame (m).

**Figure 15 fig15:**
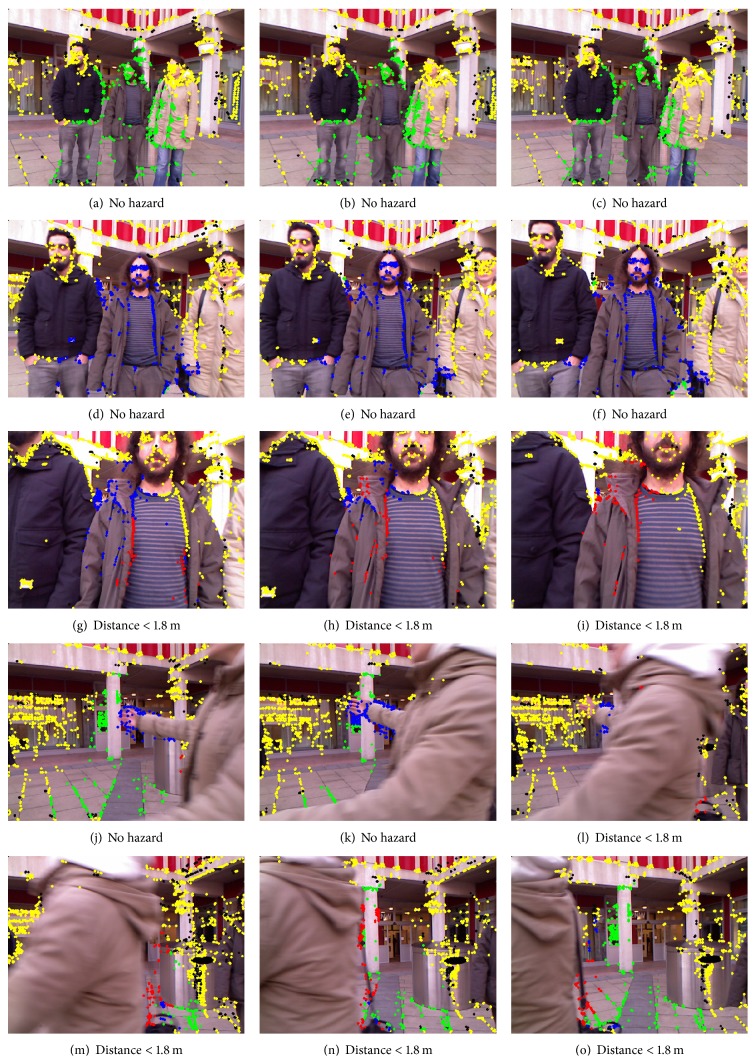
The navigation system's visual output on an outdoor video sequence with people walking around the subject (blindfolded) as dynamic obstacles. In frame (g), system starts sensing hazard shown with red points but loses it after 3 frames; therefore, no warning is issued.

The current system can process Kinect data at 8 to 10 frames per second (on an Intel Core 2 quad 2.83 GHz processor), which is fast enough to warn the trial subject of any collisions, who is walking with an average speed of 0.65 m/s (blind). Figures 13 [Fig fig14] [Fig fig15]–15 are used to show system's processing at selected frames along with simulated navigational path recorded at each location. In each of Figures 13–15, the frames in the rows show gradually decreasing distances from obstacles.

The colour of the corner points indicates whether they are potential hazards or not, using the annotation scheme described in the previous section. Each row contains a sequence of three frames of video showing a gradual decrease in distance from obstacle. The system's response at indoor locations was found to be more appropriate and better timed than in outdoor locations. This was not unexpected because strong sunlight and shadows create blind spots in the depth images captured by Kinect and these affect the system's accuracy.

Table 3 gives analysis of obstacle detection and walking speed of blindfolded and blind person. The speed of blindfolded person appeared to be less than of blind person. As mentioned before, a blindfolded person does not feel comfortable using a mobility aid and therefore walks slowly. Furthermore, the walking speed of a blind person is very close to his natural walking pace. Therefore, the system shows its potential in becoming a good navigational aid for visually impaired people.

Table 4 gives a comparison of the proposed system with previously proposed navigation systems in the last decade. Although sonar and camera are cheap sensors, however, increasing the number of sensors as in [23, 24], the systems becomes costly and needs proper calibration. Therefore, instead of using a number of similar sensors, the proposed system exploited the advantages of two cheap sensors and produced good results, a concept also used in [25, 26].

## 4. Current Limitations and Future Directions

The system is capable of identifying obstacles at reasonable distances and speeds; however, it was suggested by the blind user that if an automated navigation system can be combined with a white cane, one can have a safe and reliable mobility aid. This is mainly because surface terrain and low-level obstacles can be identified easily using a white cane, whereas an automated navigation system can help locate head-level obstacles and identify obstacles blocking one's path.

All the problems experienced with the current* system* appear to be due to the infrared sensor as it starts giving blind spots (no depth values) under strong sunlight. In the future, replacing it with some other depth sensor, such as a laser strippers, might yield more accurate responses. However, the concept of combining vision with sensed imagery proves to work well. Android mobile phones are equipped with GPS, inertial sensor, and an RGB camera, a combination that may well be worth exploring for this kind of systems.

## 5. Conclusion

A navigation system for visually impaired people has been designed, implemented, and assessed in both indoor and outdoor environments. Input from Kinect's camera and distance sensor compensates for limitations of each individual sensor.

The system was tested on both a blindfolded person and a visually impaired person. Both users found the system to be promising and highlighted its potential in becoming a good navigational aid in the future. Although some problems were experienced with the Kinect in outdoor locations, it was found to be reasonably reliable indoors. The proposed solution also provides strong justification for using hybrid technologies, because of the inability of all sensors to work under all environmental conditions (sunlight, rain, etc.).

## Figures and Tables

**Table 1:** Commonly used sensory aids for visually impaired persons.

Sensor	Cost	Type	Usability
Guide dog	High	Genesis	Not easily available; needs carer
White cane	Low	Mechanical	Slow and can only find obstacles through touch
Ultrasonic sensor	High	Electronic machine	Signal reflection or absorption may result in false information
Camera	Low	Electronic machine	Needs proper installation
Infrared sensor	Low	Electronic machine	Can only be used for short distances
Laser	High	Electronic machine	Sensitive to sunlight
GPS	Low	Electronic machine	Can only be used in outdoors
Mobile robots	High	Electromechanical	Can only be used on plain and smooth surfaces

**Table 2:** Calibration parameter values of the Kinect sensor used in this work. *f*_*x*_, *f*_*y*_ and *f*_*xd*_, *f*_*yd*_ are the focal lengths in *x* and *y* (to accommodate astigmatism of the lens systems) of the colour camera and depth sensor, respectively. *p*_1_ and *p*_2_ are radial distortion parameters, while *k*_1_, *k*_2_, and *k*_3_ are tangential distortion parameters.

*f*_*x*_ = 5.2922 × 10^2^	*t*_*x*_ = 3.2895 × 10^2^
*f*_*y*_ = 5.2556 × 10^2^	*t*_*y*_ = 2.6748 × 10^2^

*f*_*xd*_ = 5.9421 × 10^2^	*t*_*xd*_ = 3.3931 × 10^2^
*f*_*yd*_ = 5.9104 × 10^2^	*t*_*yd*_ = 2.4274 × 10^2^

*k*_1_ = 2.6452 × 10^−1^	*p*_1_ = −1.9922 × 10^−3^
*k*_2_ = −8.3991 × 10^−1^	*p*_2_ = 1.4372 × 10^−3^
*k*_3_ = 9.1192 × 10^−1^	

**Table 3:** Obstacle detection rate and comparison of blind and blindfolded persons.

	Blindfolded		Blind	
Obstacle	Detection	Speed	Detection	Speed
	time (sec)	(m/sec)	time (sec)	(m/sec)
Wall (corridor)	0.25	0.4	0.25	0.6
Pillar (open square)	0.3	0.3	0.3	0.5
People (open square)	0.28	—	0.25	—
Noticeboard	0.3	0.3	0.3	0.5
Obstacles (lab)	0.25	0.3	0.25	0.4
No obstacle (open square)	0.25	0.4	0.25	0.55

**Table 4:** Comparison with navigation systems developed in the last decade for visually impaired people.

References	Year	System	Cost	Accuracy	Testing				Limitations
					Indoor/outdoor	Tested by	Test	Feedback	
[23]	2003	NavBelt and the GuideCane	High	Good	Both	Blindfolded	Obstacle avoidance and natural walking	Binaural feedback system and haptic feedback	Ultrasonic sensor cannot detect all objects under all conditions, NavBelt is slow but GuideCane is fast; however, a number of sensors need more power.

[24]	2007	Ultrasonic sensor	Medium	Low	Indoor	Blindfolded	Task completion	Vibrotactile	Needs some surface for producing distance output; sound waves get absorbed in some surfaces like sponge, cloth, skin, and so forth.

[25]	2011	Hybrid ultrasonic, camera, and eBox	Medium	Indoor		Blindfolded	Obstacle avoidance and recognition	Voice	Not tested in outdoors, difficult to manage multiple sensors. Ultrasonic sensor has limitations of giving false data for some surfaces discussed before.

[27]	2012	Wearable Navigation System using SLAM	Medium	Medium	Both	Blindfolded	Path following	Verbal feedback	Complex computation for SLAM using landmarks.

[28]	2012	Electronic cane	High	Good	Indoor	Blindfolded	Obstacle avoidance	Verbal feedback	Five sensors are integrated to gather obstacles' data; therefore, malfunctioning of any one of them can cause system failure.

[29]	2013	Smartphone based navigation assistance	Low	Medium	Outdoors	Blindfolded	Obstacle avoidance	Verbal feedback	Training data is required and robust obstacle detection is required.

[26]	2013	Hybrid, Infrared, and ultrasonic	Low	Both		Blindfolded	Obstacle avoidance	Vibrations	Infrared sensor is unable to work under strong sunlight; similarly, ultrasonic sensors get effected by loud environmental noises such as hissing sound produced by air houses.

[30]	2015	Robot Guide	High	Medium	Indoor	Blind	Natural walking	Verbal feedback	A big robot that needs space for its own movement can only walk on smooth surfaces.

[8]	2015	Infrared sensor	Low	Low	Indoor	Blindfolded	Travel time	Melodies	Infrared sensor alone is not reliable because of its blindness under strong light and rough surfaces.

Our system	2015	Combined vision techniques and depth map from Kinect	Low	Good	Both	Blind and blindfolded	Obstacle avoidance and walking in natural environment	Verbal feedback	Infrared sensor is unable to work under strong sunlight.
